# Medial Abrasion Syndrome: A Neglected Cause of Knee Pain in Middle and Old Age: Erratum

**DOI:** 10.1097/01.md.0000466520.95099.b7

**Published:** 2015-05-29

In the article “Medial Abrasion Syndrome: A Neglected Cause of Knee Pain in Middle and Old Age”,^1^ which appeared in Volume 94, Issue 16 of *Medicine*, Table 3 was incorrectly formatted. The issue has since been corrected online.
